# Analytical Ultracentrifugation-Calibrated Anion-Exchange Chromatography for Sensitive and Intact Determination of Osteopontin in Infant Formula and Dairy Products

**DOI:** 10.1021/acs.jafc.3c03589

**Published:** 2023-09-05

**Authors:** Xiangxin Wang, Dongying Cui, Xueyin Qu, Hong You, Fan Lei, Jianqiao Li, Yang Xie, Haoshu Zhang, Yongjiu Zhang, Shilong Jiang, Qinggang Xie

**Affiliations:** † Heilongjiang Feihe Dairy Co., Ltd., Beijing 100015, China; ‡ China Excellent Milk Academy (Tianjin) Co., Ltd., 300400 Tianjin, China; § Eurofins US Food, 2200 Rittenhouse St Ste 175, Des Moines, Iowa 50321-3155, United States; ∥ State Key Laboratory of Membrane Biology, Tsinghua University, Beijing 100084, China

**Keywords:** osteopontin, anion-exchange chromatography, analytical ultracentrifugation, polarity-reversed capillary isoelectric focusing, infant formula

## Abstract

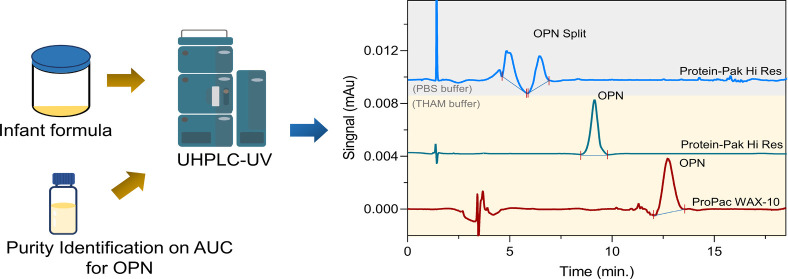

Osteopontin is a crucial protein ingredient that has been applied in fortified dairy products and infant formula. It has great significance to infant gut health and brain development. However, current techniques including enzyme-linked immunosorbent assay and liquid chromatography coupled with mass spectrometry are still facing the bottleneck of low sensitivity and indirect quantification. Moreover, the unavailable certified commercial OPN standard hinders its accurate quantification. Herein, a novel method of anion-exchange chromatography was established to determine OPN concentration in several dairy matrices. The polarity-reversed capillary isoelectric focusing was utilized to measure the exact isoelectric point (pI) to support method development for OPN separation. Analytical ultracentrifugation was used to calibrate the purity of intact OPN to develop an in-house reference standard. The method showed the merits of limits of detection to 0.04 mg/100 g, relative standard deviation of reproducibility <5% for 13 out of 14 tested matrices, and an average recovery rate of 101.3%. This method has shown the potential to be adopted as an international standard method for the quantification of intact OPN in infant formula and dairy products.

## 1 Introduction

OPN (Osteopontin) is an acidic and highly phosphorylated glycoprotein with an open and flexible structure consisting of two parts (full length and N-terminal fragment).^1−3^ OPN is involved in various physiological functions (such as ectopic calcification inhibition,^4,5^ bone remodeling,^6,7^ and immunoregulation),^8,9^ playing a vital role in infant gut health and brain development. Currently, bovine OPN has been considered a potential candidate protein ingredient in infant formula to mimic breast milk because it shares high structural similarity compared to human OPN.^10,11^ Therefore, dairy-based infant formulas with fortified OPN have been attracting more attention from researchers and industrial practitioners.^12^ Nevertheless, according to the suggestions to European Food Safety Authority (EFSA) Panel on nutrition,^13^ the maximum concentration of fortified OPN should be controlled under 151 mg/mL in ready-to-eat (RTE) products. Hence, establishing an effective, reliable, accurate, and high-throughput method for the analysis of the OPN is the guarantee of the quality of dairy products and their raw materials.

Enzyme-linked immunosorbent assay (ELISA)^14,15^ and liquid chromatography coupled with mass spectrometry (LC-MS)^16,17^ are the two analytical approaches to determine the OPN concentration in dairy products. ELISA has been mostly applied in the detection of human OPN in breast milk because of its easy procedures. There are commercial monoclonal antibodies to human, mouse, and rat OPN, but some of them cross-react with bovine OPN, which leads to overestimated results.^15^ LC-MS is a sensitive and general method for the analyte determination. However, it is difficult to quantify the intact OPN directly. Only after the two parts of OPN are enzymatically digested to specific peptides,^16,18^ the OPN content can be determined by analyzing the concentrations of two portions (full length: 262 amino acids, 33.9 kDa; and N-terminal fragments: 150 amino acids, 19.8 kDa). Additionally, during the industrial processing of dairy products, the lysin could react with lactose by Maillard reaction.^19,20^ Thus, the active site of trypsin may lose specificity, and the detected peptides would be reduced, leading to a lower quantitative result. Moreover, the subjective selection of peptide ratios and the incompletion of enzymatic digestion could bring conceivable deviation in quantification.^18^

Unfortunately, although several OPN materials are commercially available (e.g., Sigma Product# O3514, SRP3131, and O4264), they were not produced as certified reference standards with adequate Certificates of Analysis to support the accurate quantification of OPN. If those reagent-grade OPN materials are used with an assumption of 100% purity, the result will be overestimated. A rapid and easy HPLC-UV method for intact OPN analysis was presented in a recent paper.^21^ However, it showed several critical flaws: (1) It used one of the above Sigma materials as its reference standard, which might lead to inaccurate quantification. (2) It failed to show chromatographic baseline separation between the OPN and other interferences from the infant formula matrix and also did not present any other method specificity or selectivity tests to prove the purity of the targeted OPN peak. (3) Only instrument precision, not method precision, was presented in its “precision study”, which only involved replicated injections of a single sample without multiple independent preparations. (4) In the accuracy study, only duplicate preparations were conducted for each level, which means it lacked sufficient statistical power to accurately measure %RSD values. (5) More importantly, we collaborated with Waters Corporation to conduct six experiments with different modifications based on this method, but we could not successfully reproduce its results and chromatograms (data can be provided upon request). Therefore, considering the complexity of the dairy matrix (i.e., fats, carbohydrates, and interferential proteins) and strictness of infant formula regulations, it is critical to develop a new method with a robust and practical process to characterize an OPN reference standard and subsequently use it to quantify intact OPN in target dairy matrices.

In this paper, a novel method based on anion-exchange chromatography was established for the determination of the OPN concentration. The analytical ultracentrifugation (AUC) was used to develop an in-house OPN reference standard by calibrating the purity of the intact OPN protein without complex hydrolysis procedures to produce peptides. In addition, the polarity-reversed capillary isoelectric focusing (cIEF) approach was utilized innovatively to measure the isoelectric point (pI) of the OPN to perform anion-exchange chromatography. To improve the OPN extraction rate, multiple conditions and processing sequences in the pretreatment were screened and the impurities precipitation was accelerated by the releasing agent CaCl_2_. Based on the method validation study results, we believe that our OPN method could be used to establish standards in the food industry and applied to direct quantification of intact OPN protein for dairy products, including infant formula.

## 2 Materials and Methods

### 2.1 Reagents and Chemicals

Sodium chloride (NaCl), trifluoroacetic acid (TFA), lactic acid, calcium chloride (CaCl_2_), hydrochloric acid (HCl, 36%), acetonitrile (ACN), urea (NH_2_CONH_2_), aminodiacetic acid (HN(CH_2_COOH)_2_), ammonia solution (NH_4_OH, 25%), methyl cellulose (MC, viscosity: 1500 cP), and sodium hydroxide (NaOH) were all purchased from Sigma-Aldrich (USA). Acetic acid and phosphoric acid (85 wt %) were brought from Merck (USA); sinapic acid (SA) was provided by Supelco (USA). The amphoteric electrolytes: Pharmalyte 3–10 carrier ampholytes were purchased from Cytiva (USA). The pI markers (pH = 5.500 and 3.210) were purchased from AB Sciex (USA) and AES (Canada). The tris(hydroxymethyl)aminethane (THAM) solution of 1 M was purchased from Thermo Fisher (USA). Lactoferrin (L9507), α-lactalbumin (L5385), β-lactoglobulin (L3908), bovine serum albumin (BSA), α-casein (C6780), β-casein (C6905), κ-casein (C0406), and lysozyme (L6876) were all purchased from Sigma-Aldrich (USA). Casein glycomacropeptide (CGMP) was provided by Agropur (BiPRO GMP 9000). Casein phosphopeptides (CPP) were provided by Ingredia Nutritional (OSTEUM CPP). All of the reagents were directly used for experiments without further purification.

The cIEF gel was obtained by mixing 15 mL of ultrapure water and 0.4 g of MC powder, followed by stirring for 5 min at 80 °C. After the mixture was removed from the heater, an ice–water mixture was added to the solution to 40 mL. Then, the solution was stirred every 30 min until cooling to −20 °C. Then, the obtained mixture was stored at 4–8 °C overnight. Furthermore, 3 M urea gel solution was prepared by dissolving 1.8 g of urea and 6 mL of cIEF gel with ultrapure water added, meeting a final volume of 10 mL. After mixing, the as-prepared solution was stored at 4 °C.

### 2.2 Sample Resources

To develop an in-house reference standard, a high-purity OPN protein ingredient was extracted and provided by Arla Foods Ingredients (Denmark). The raw cow’s milk (origin: Kedong, Heilongjiang Province, China), raw goat’s milk (origin: Long, Shaanxi Province, China), and commercially available infant formula powders with or without the OPN were supplied by Feihe Dairy Co. Ltd. (China). All the infant formulas mentioned above were formulated and prepared in accordance with relevant requirements of *Codex Stan 72–1981 Standard For Infant Formula And Formulas For Special Medical Purposes Intended For Infants (Amended in 2015)* and *CODEX STAN 156–1987 STANDARD FOR FOLLOW-UP FORMULA (Amended in 2017)*.

### 2.3 Development and Characterization of an In-House OPN Reference Standard

UV spectra of OPN from 190 to 300 nm at 10 mg/L in water were collected to verify the purity of the OPN primarily by Agilent Cary 60 UV spectrophotometer (USA), as shown in Figure S1. The accurate purity of the in-house OPN reference standard was calibrated by an analytical ultracentrifuge (Optima AUC, ECKMAN COULTER, USA), equipped with four-well rotor 60 Ti two sample cells and a sapphire window.^22^ Briefly, high-purity OPN powder was dissolved in mobile phase A (20 mM THAM in 10 mM NaCl, pH 8.00), resulting in an OPN concentration of 1 mg/mL. After cleaning the window and other accessories, 380 μL of the obtained solution was added. Then, the sample cells were assembled into the AUC instrument. The blank control sample was prepared by adding 380 μL of mobile phase A. The temperature was set at 20 °C, and the rotational speed was 50,000 rpm. The UV-absorbance signals at 280 and 260 nm were collected by Nanodrop (NANODROP ONE, Thermo Fisher Scientific, USA). This in-house OPN standard’s protein content of the in-house OPN standard was calibrated in six replicates. The interference data were acquired at an interval of 70 s until the sedimentation process was completed after 14 h.

The normalized content was calculated by GUSSI software, where the 137 experimental data points were processed using Sedfit 14p81 software. Typically, the parameters were set as follows: resolution = 200, *S*_min_ = 0, *S*_max_ = 25, buffer density = 1.0000, buffer viscosity = 0.01002, partial spec. volume = 0.73. Radial-invariant (RI) and time-invariant (TI) noise subtractions were applied. The meniscus position was allowed to float, allowing the software to automatically choose the optimal position. The confidence level (F-ratio) was set as 0.68, and the frictional ratio was 2.7.

The purity result of the in-house OPN reference standard was then verified by the mass balance calculation based on size exclusion chromatography (Waters ACQUITY Arc Bio UHPLC, USA), repeated 10 times, and the results are listed in Table S1. Approximately 100 mg of the OPN was weighed accurately and dissolved in 5 mL of water, mixed by vortex. Subsequently, the mixture was analyzed on the chromatography system with the TSK gel UP-SW3000 column (4.6 × 300 mm, 2 μm, 3000 Å, TOSOH, Japan). The mobile phase was 50 mM NaH_2_PO_4_ in 300 mM NaCl, pH = 7. The flow rate was fixed at 0.25 mL/min, injection volume was 10 μL, and column temperature was 30 °C. Chromatographic purity was calculated by a UV detector at 220 nm by peak area normalization.

### 2.4 Capillary Focusing Method

To support anion-exchange chromatographic method development, polarity-reversed cIEF measurements were performed on a SCIEX PA 800 Plus Capillary Electrophoresis System coupled to a UV detector. The data were acquired and analyzed by 32 Karat software. The 50 μm fluorocarbon polymer-coated capillaries (Agilent, USA) with a total length of 30.2 cm were used for separation. The operation temperature was fixed at 25 °C, and the samples were stored at 10 °C.

The samples to be tested were prepared by mixing 10 μL of an OPN protein solution (20 mg/mL), 180 μL of a 3 M urea-gel solution, 12 μL of 3–10 carrier ampholytes, 30 μL of an anodic stabilizer (200 mM iminodiacetic acid), and 1 μL of pI markers (pH 3.21 and pH 5.50). Then the mixture was vortexed for 30 s before analysis.

The capillary tubes were rinsed with 350 mM acetic acid, water, and cIEF gel at 50 psi for 5, 2, and 5 min, respectively. Before the samples were injected, urea (6.8 M) and water were applied to flush the capillary for 3 and 2 min at 50 psi, respectively. Then, each capillary was slowly filled with a sample under a pressure of 25 psi for 99 s. Meanwhile, 200 mM phosphoric acid and 300 mM NaOH were employed as the anodic and cathodic solutions, correspondingly. After the samples were focused for 15 min at −25 kV, the cathodic solution was replaced by 100 mM NH_4_OH solution, and the focused proteins were migrated to the detecting window under the voltage of −30 kV with a detection channel of 280 nm. In this way, the isoelectric point of the OPN was calculated according to the linear relationship between the theoretical isoelectric points of the pI markers and their corresponding migration durations.

### 2.5 Chromatographic Separation Condition

The OPN analysis based on chromatographic separation was performed on an ultrahigh performance liquid chromatography (UHPLC) system (Waters ACQUITY Arc Bio UHPLC, USA) coupled with a diode array detector (DAD). Two ion-exchange columns were applied as follows: Protein-Pak Hi Res Q (Waters, 5 μm, 4.6 × 100 mm); ProPac WAX-10 BioLC Analytical (Thermo Fisher Scientific, 10 μm, 4.0 × 250 mm). The working temperature of the columns was fixed at 40 °C, and the autosampler was controlled at 10 °C. The UV wavelength was chosen as 220 nm for detection. The injection volume was set as 10 μL, and a 0.1% (v/v) TFA aqueous solution was used as the needle-washing solution. The chromatogram data were acquired and analyzed by Empower 3 software. Gradient elution was carried out with a THAM buffer system at a flow rate of 0.4 mL/min. Mobile phase A was 20 mM THAM in 10 mM NaCl, pH 8.00; mobile phase B was 20 mM THAM in 800 mM NaCl, pH 8.00. A typical elution gradient was set as followings: for mobile phase B, 0–3 min, 50%; 3–6 min, 50–80%; 6–10 min, 80–90%; 10–13 min, 90%; 13–14 min, 90–50%; 14–20 min, 50%.

### 2.6 Peak Identification by MALDI-TOF-MS

OPN was purified and fractionated by a Waters UHPLC-DAD system equipped with a Fraction Manager. A typical procedure was as follows: the matrix of Sinapic acid (10 mg/mL) was diluted in a mixed solvent (acetonitrile: water: trifluoroacetic acid = 7:3:0.01, v/v/v). Then, the OPN fraction was collected according to the corresponding retention time. Then, the obtained fraction was mixed with the matrix in a 1:1 ratio (v/v). Furthermore, 1 μL of the mixture was dropped on a target plate and dried at room temperature to be tested by matrix-assisted laser dissertation ionization-time-of-flight mass spectrometry (MALDI-TOF MS).

MALDI-TOF MS analysis was performed on a Bruker ULTRAFLEX mass spectrometer (USA). The mass spectra were acquired by a SmartBeam laser (355 nm) operated at 200 Hz with a laser focus of 50 μm. Data were collected in linear mode with a target plate voltage of 19 kV and processed using DateAnalysis 3.0 software (Bruker Daltonics).

### 2.7 Sample Preparation Optimization and Final Method

To optimize sample preparation, first, the combination of temperature was screened. The samples were heated and ranged from 50 to 100 °C with duration of 10–60 min. A total of 121 experimental conditions were assessed. Based on this optimized combination of temperature and time, the amount of CaCl_2_ was further investigated. The amount of calcium chloride added starts from 0.5 mL; for every 0.5 increments, a total of 36 experimental conditions were screened with 2 repetitions. A heat map was generated to summarize and demonstrate the results.

**Figure 1:**
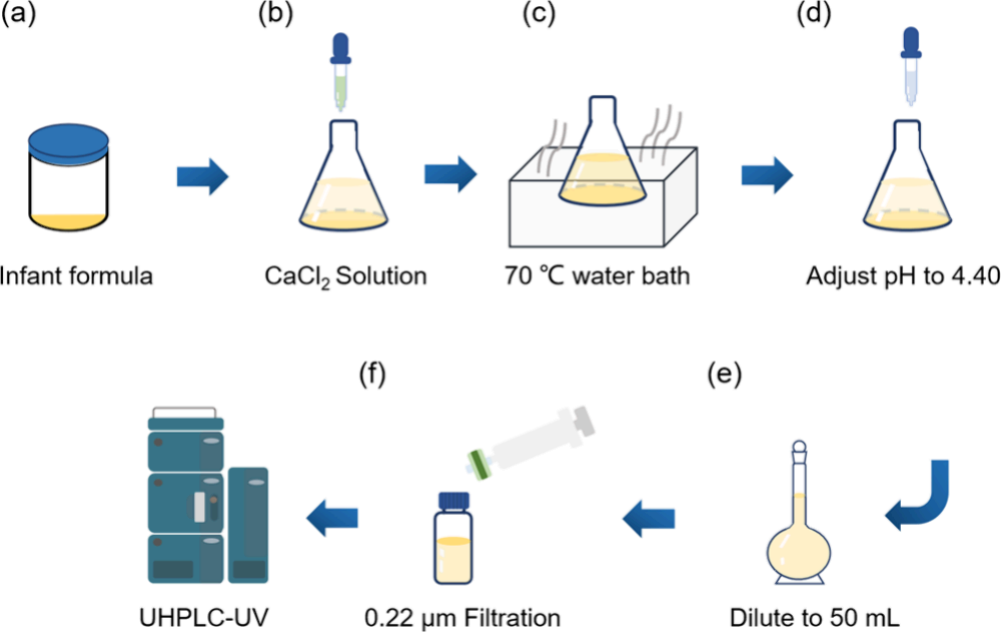
Schematic illustration of sample preparation procedure: (a) milk powder dissolved in 40 °C water, (b) addition of CaCl_2_ solution, (c) 70 °C water bath for 20 min, (d) adjusting the pH, (e) sample dilution to 50 mL, and (f) sample filtration for UHPLC-UV analysis.

As shown in Figure 1, 5.0 g of milk powder was dissolved into 30 mL of warm water at 40 °C (or 30 mL liquid milk was directly warmed) and vortex-mixed for 5 min. Then, 4 mL of 500 mM CaCl_2_ solution was added into the mixture, and the sample was incubated in a 70 °C water bath for 20 min. After removing the sample from the water bath and cooling it to room temperature, the solution pH was accurately adjusted to 4.40 with lactic acid (10%, v/v) and further diluted to 50 mL. Finally, the solution was filtered by a 0.22 μm membrane into the vial for UHPLC analysis.

### 2.8 Final Method Validation

The anion-exchange chromatographic method was systematically investigated by evaluating the analytical performance such as system suitability, linearity, sensitivity, specificity, precision, accuracy (recovery rate), and stability. Standard solutions at different OPN concentrations in the range of 10–500 mg/L were used to establish the standard curve for the regression equation. In brief, a stock solution of 1 mg/mL of OPN was prepared by carefully weighing and dissolving the standard in mobile phase A. The accurate concentration was calculated according to the purity of the OPN measured by AUC. The series of working solutions (10, 25, 50, 75, 100, 250, and 500 μg/mL) were diluted by the stock solutions of the OPN in mobile phase A. The limits of detection (LOD) and quantification (LOQ) were assessed by analyzing the response conditions at the signal-to-noise ratio (S/N) values of 3 and 10, respectively. The system suitability and stability were tested by analyzing a standard solution at 25 mg/L concentration. The precision of this method was assessed by analyzing 14 dairy product samples on three different days. The accuracy (recovery rate) of this method was investigated through the standard addition method by spiking OPN standards into an infant formula sample 22T034 at three levels (25, 75, and 250 mg/100 g; *n* = 10). The equation of recovery rate calculation is listed below:
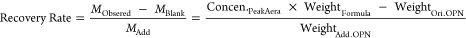
where Concen._Peak Aera_ is observed concentration of OPN in formula, mg/100 g; Weight_Formula_ is the weight of formula powder; g; Weight_Ori. OPN_ is the original OPN content in formula, mg/100 g; Weight_Add. OPN_ is the additional OPN content by manual addition to formula, mg/100 g.

A total of 14 real samples with different matrices were evaluated by repeating the preparation on 3 consecutive days.

## 3 Results and Discussion

### 3.1 Calibration of an In-House OPN Standard by AUC

**Figure 2:**
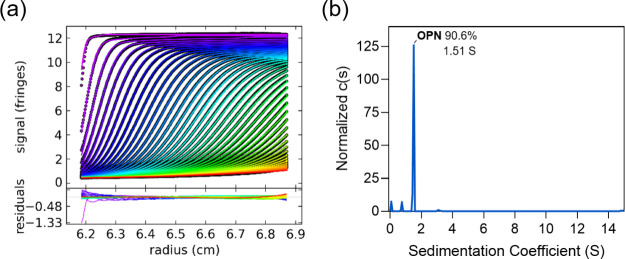
(a) Sedimentation velocity analysis of OPN; original data (top) and residuals plot (bottom); (b) distribution of sedimentary species for the commercial OPN standard.

In the AUC experiment, the target component particles would exhibit continuous sedimentation during high-speed centrifugation. By UV and laser light radiation, the AUC instrument could collect optical interferometric signals to calculate the physical and chemical properties of the target particles, such as the sedimentation coefficient, molecular weight, particle shape, material distribution, and content purity.^23^ By analyzing the AUC results of the 280 nm UV and laser light, the presence of nonprotein impurities in the OPN standards was discovered. Consequently, the interferometric results could be used for quantification. As shown in Figure 2a, the root-mean-square deviation (rmsd) was 0.047, within the confidence range. The obtained friction coefficient was 2.76, indicating a rodlike shape of the OPN. As displayed in Figure 2b, the sedimentation coefficient was 1.51 S. The calibrated purity of the OPN standard was 90.6 wt % (dry basis), which was consistent with the mass balance calculation, as shown in Figure S2 and Table S1.

Meanwhile, when changing the buffer system from 20 mM THAM to 20 mM PBS, the OPN bands of tetramers and 8-mers were found (Tables S2 and S3).^24^ It suggested that this buffer system (20 mM THAM, 10 mM NaCl, pH 8.00) was an adequate condition for the analysis of OPN.

### 3.2 Measurement of the pI of OPN

**Figure 3:**
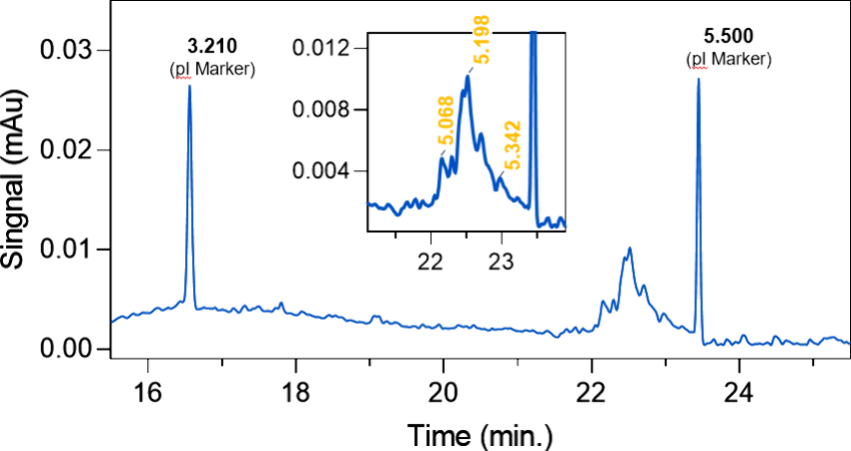
Typical electropherogram of OPN separated by the polarity-reversed cIEF method.

Since environmental pH determines the surface charge of a protein, it can greatly affect chromatographic behavior. Therefore, before developing the analysis methodology for a specific protein, it is necessary to measure the corresponding pI. As previously reported, OPN is an acidic protein with a theoretical pI of 4.30.^25^ In another method based on the amino acid sequences without posttranslational modification, the pI was theoretically calculated to be 4.46.^26,27^ To obtain an accurate pI of the OPN, an improved cIEF method was established and validated in this study. The polarities of the cIEF mode were reversed, in which the left electrode was negatively charged while the right was positive. A weak basic aqueous solution of 100 mM NH_4_OH was employed as a chemical migration reagent. It can be expected that the acidic proteins would move faster than the basic ones to the detection window during the migration process, indicating that the acidic OPN could have a shorter migration time and produce better reproducibility. As the result shown in Figure 3, the pI of OPN measured was distributed in the range of 5.07–5.34. The relatively broad pH spanning of 0.27 units suggested that the intact OPN sample might consist of phosphorylation and glycosylation sites with multiple dissociable amino acid residues and phosphoric residues in the flexible spatial structure.^28^

### 3.3 Establishing the Anion-Exchange Chromatography Method

Taking advantage of separating different net charged components at a specific pH, the anion-exchange chromatography with quaternary amine groups was utilized for the analysis of OPN. According to the Henderson equation,^29^ the pH of the mobile phase should be adjusted to two pH units above the pI of the protein. Thus, over 99% of the proteins could be negatively charged, which would enable electrostatic adsorption of the positively charged −CH_2_N^+^(CH_3_)_3_ group on the column. Since the measured pI of OPN was in a range of 5.07–5.34; hence, the pH of the mobile phase should be over 7.34. Practically, the final pH was set as 8.00.

**Figure 4:**
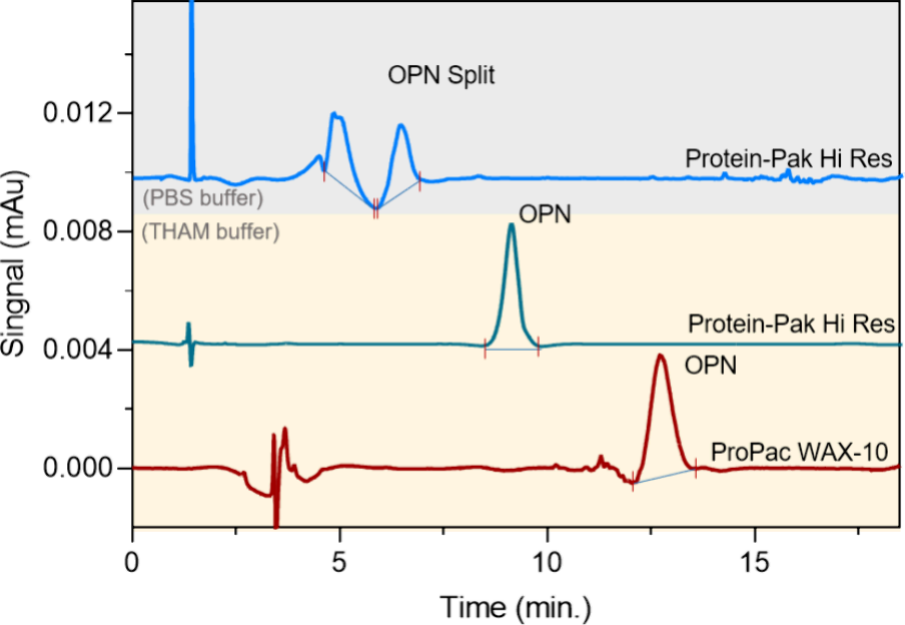
Comparative chromatograms under two different buffer conditions (gray area: PBS, and yellow area: THAM) and THAM performance under two columns Protein-Pak Hi Res (green line), and ProPac WAX-10 (red line).

First, the commonly used 20 mM PBS was chosen as the buffer system. As shown in Figure 4 (upper part), split peaks of the OPN standard were observed, indicating that the PBS would change the charge distribution of OPN, which was consistent with the AUC results. Then, the THAM buffer system with Cl^–^ as the counterion was employed as the mobile phase. Two anion-exchange columns (Protein-Pak Hi Res Q and ProPac WAX-10 BioLC) were applied to screen the conditions. The results presented in Figure 4 indicated that the Protein-Pak column could perform a sharp and symmetric peak shape of the OPN with excellent repeatability, owing to the multilayer network in stationary phase particles providing a more distributional surface. In contrast, the obtained OPN peak from the ProPac WAX-10 column displayed was wider, due to the nonporous particles, whose separation was only by the diffusion on the particle surface. Therefore, the Protein-Pak Hi Res Q column could afford higher protein load and improve separation efficiency, enabling analysis of complex biomolecules in a short window.

### 3.4 Screening for Sample Preparation Conditions

**Figure 5:**
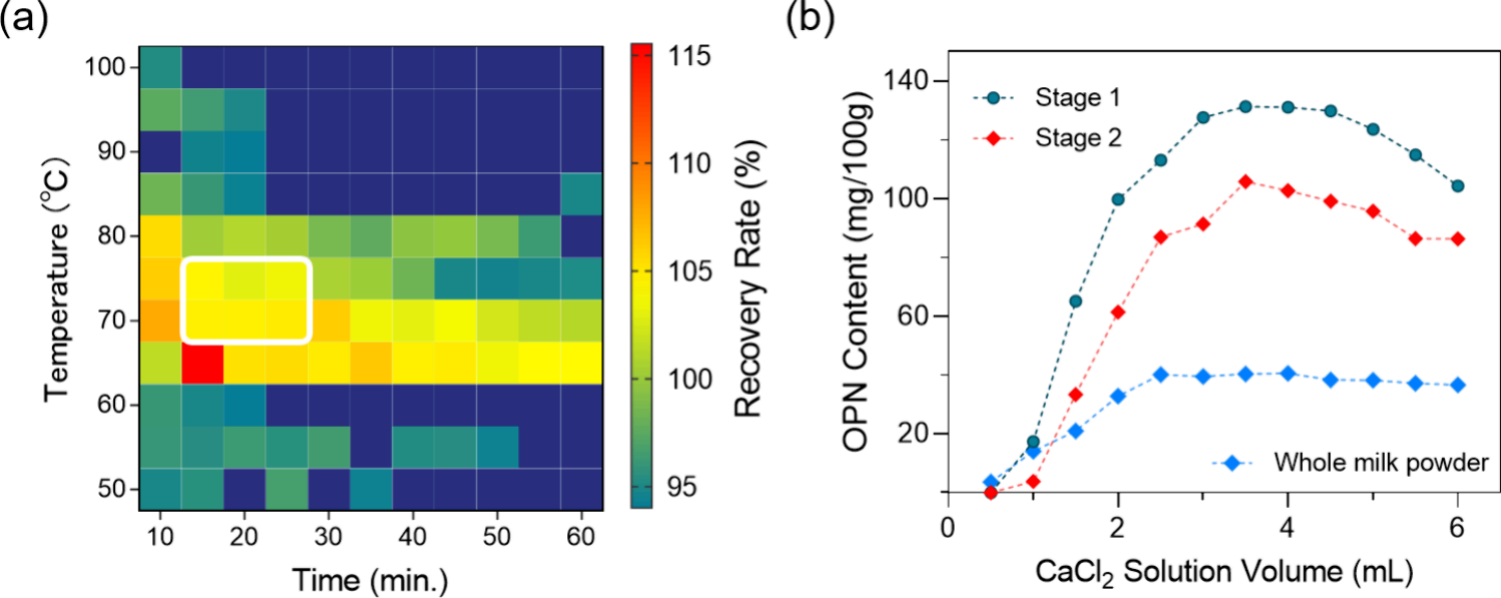
(a) Heatmap presentation of the OPN recovery rate under different extraction temperatures and time combinations. (b) Plots of OPN content with 0.5–6 mL 500 mM CaCl_2_ added in stages 1 and 2 as well as whole milk powder.

To study the effects of temperature and time on the extraction rate (recovery) of the OPN, the temperature range was set as 50–100 °C, and the heating duration was selected in the range of 10–60 min. As shown in Figure 5a, an orthogonal set of procedures and conditions were screened to obtain the optimal pretreatment conditions. It was found that when the temperature was above 80 °C, and the heating time was over 25 min, a low content of the OPN was observed. When the extraction temperature is low and the extraction time is short, the extraction condition might not fully destroy the interaction between OPN and casein, resulting in the prohibition of access of the OPN for analysis and subsequently a low recovery rate. In other conditions with a high extraction temperature, we found low OPN recovery rates regardless of the extraction duration. We speculated that the high temperature might have caused protein denaturation. Briefly, the calculated recovery rates of the OPN were close to 100% when the temperature was in the range of 70–75 °C and the duration was 15–25 min. Taking the reproducibility and experimental efficiency into consideration, the most suitable condition was set in a 70 °C water bath for 20 min.

Additionally, Ca^2+^ could be used as a releasing reagent for the OPN and could precipitate certain impurities such as casein. As shown in Figure 5b, with the addition of CaCl_2_, the recovery rate of the OPN increased initially. However, when the added volume was over 4.5 mL, the recovery rate began to decrease. We speculate that CaCl_2_ might have triggered competitive adsorption, which would block the electrostatic interaction between negatively charged carboxylate residues (aspartic or glutamic acid) in the OPN and casein micelles. As a result, the optimum pretreatment condition was eventually chosen as heating at 70 °C for 20 min with the addition of 4 mL of CaCl_2_ (500 mM) as a releasing agent. More experiments are needed in the future to elucidate and prove the mechanism behind this optimal pretreatment condition.

### 3.5 Optimization of the Sample Preparation Procedure

To further optimize the sample preparation conditions and investigate the extraction mechanism, several pretreatment procedures were tested, and the corresponding recovery rates are listed in Table 1. It evidenced that all the listed critical pretreatment steps (including pH adjustment, heating, and CaCl_2_ addition) played important roles and interplay with each other in OPN recovery rates. In condition No. 1, the added Ca^2+^ would change the charged state of precipitated casein micelles, leading to exposure of more negatively charged phosphoryl groups, which would attract proton and increase the pH up to 6.50. Simultaneously, the precipitated casein micelles were disintegrated into β-casein, α_s1_-casein, α_s2_-casein, and κ-casein.^31^ The OPN might have coprecipitated with Ca^2+^ under neutral conditions, resulting in an average OPN recovery rate of only 1.9%. In condition No. 2 (in the absence of CaCl_2_), the electrostatic interaction between casein and OPN was not destroyed, resulting in a limited amount of the OPN release. As for conditions 3–5, the results were attributed to the precipitation of the OPN with Ca^2+^ in the neutral environment, leading to about 50% loss of the OPN. In condition 7, the high recovery rate of 114.4% indicated that other interference factors would be dispelled during the heating process. In addition, no significant discrepancy was found among lactic acid, HCl, or HAc for pH adjustment.

**Table 1:** Recoveries of Different Pre-Treatment Sequences (*n* = 6)

condition no.	order	recovery rate (%)	RSD (%)
1	adjust pH → add CaCl_2_ → heat and cooling	1.9	11.2
2	heat and cooling → adjust pH	7.3	10.3
3	adjust pH → heat and cooling → add CaCl_2_	41.0	15.4
4	add CaCl_2_ → heat and cooling	43.6	12.4
5	adjust pH → add CaCl_2_	56.1	8.7
6	add CaCl_2_ → heat and cooling → adjust pH	100.5	4.2
7	add CaCl_2_ → adjust pH	114.4	12.5

By analyzing the results in Table 1, we found that the treatment combination of condition No. 6 was optimal. In that case, an appropriate amount of Ca^2+^ would bond to casein, resulting in breaking the electrostatic effect inside the micelles and releasing the peptides of the OPN that was originally linked to casein. In this procedure, the Ca^2+^-sensitive proteins (such as β-casein, α_s1_-casein, and α_s2_-casein) were precipitated simultaneously; thus, the samples were purified. Moreover, when the system was heated, these proteins could form disulfide bonds with κ-casein and coprecipitate at pH 4.40,^32^ which further reduced the risk of column contamination in UHPLC. The diluent pH value was therefore designed to be 4.40 to avoid the Ca^2+^-facilitated OPN precipitation after cooling and lead to further precipitation of casein.

### 3.6 Validation of the Methodology

#### 3.6.1 Method Specificity and Selectivity

**Figure 6:**
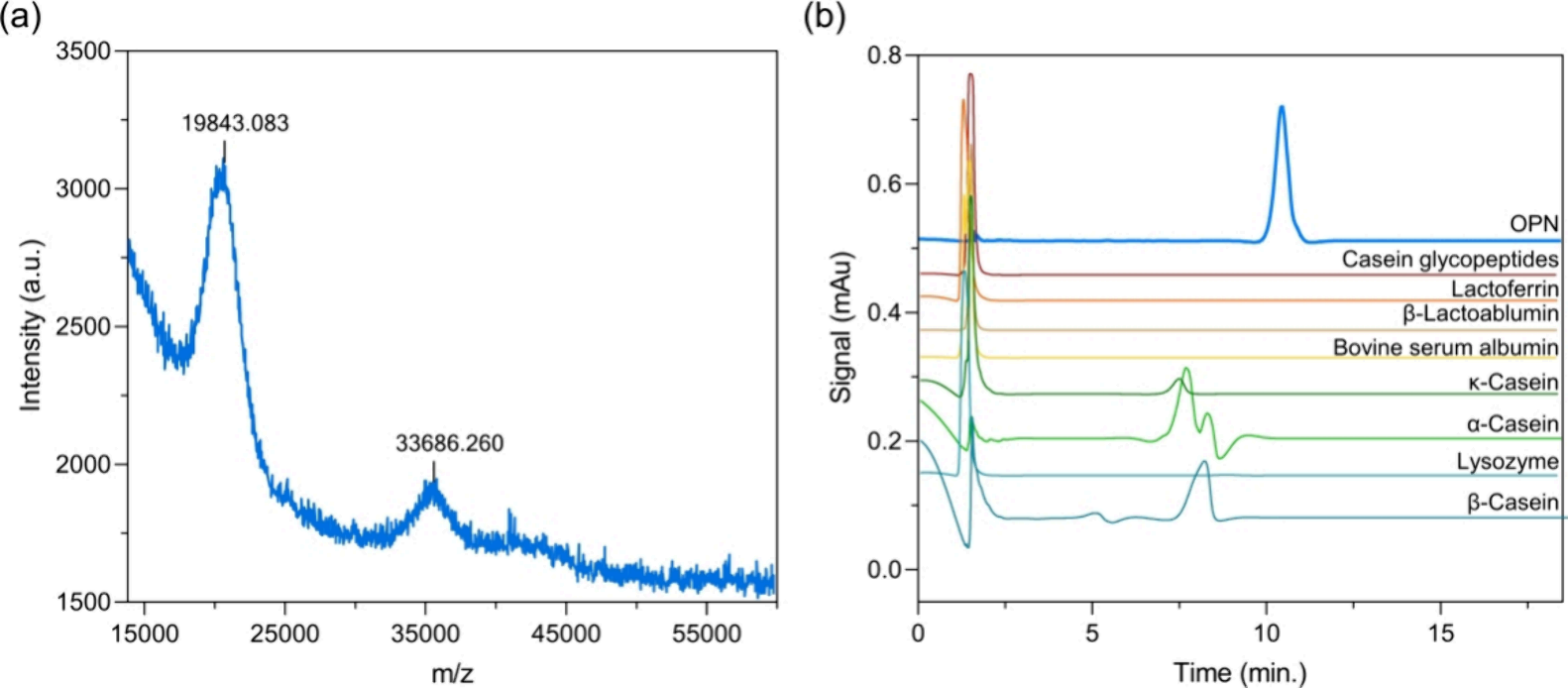
Specificity and selectivity. (a) MALDI spectrum of OPN fraction and (b) test on the possible interference proteins in the dairy product.

As shown in Figure 6a, the target OPN peak was collected by the fraction manager and identified by MALDI-TOF-MS. It revealed that the OPN was composed of two components: full-length OPN and an N-terminal fragment. The full-length OPN had a molecular weight of ∼33.7 kDa, including amino acids (29.3 kDa), phosphorylations (∼1.7 kDa), and O-linked glycosylations (2.9 kDa). The most abundant N-terminal fragment showed a molecular weight of ∼19.8 kDa, including 16 kDa of amino acids, 0.9 kDa of phosphorylations, and 2.9 kDa of O-linked glycosylations. Our MALDI-TOF experiment was conducted to successfully generate data to match the OPN molecular weight information described in Generally Recognized as Safe (GRAS) certification (GRN 716, Bovine milk osteopontin).^12^

Then, the main protein components from milk, such as α-lactalbumin, β-lactoglobulin, bovine serum albumin (BSA), lactoferrin, casein glycomacropeptide, α-casein, β-casein, κ-casein, lysozyme, and casein phosphopeptides, were chosen for the selectivity assessment. After being dissolved in mobile phase A, these samples were analyzed under the same chromatographic conditions. As shown in Figure 6b, lactoferrin, α-lactalbumin, β-lactoglobulin, bovine serum albumin (BSA), casein glycomacropeptide (CGMP), and lysozyme all eluted at dead time, indicating that these proteins are not retained under these chromatographic condition. The peaks of α-casein, β-casein and κ-casein were eluted before OPN without interferences, demonstrating an excellent selectivity of this method.

#### 3.6.2 System Suitability and Stability

The system suitability was investigated by evaluating the repeatability and stability of the 25 mg/mL OPN standard solution. The repeatability of the UHPLC system showed excellent RSDs of 0.46 and 0.71% in peak areas and heights, respectively, as shown in Tables S4 and S5. The stabilities of the 25 mg/L OPN standard at intervals of 0, 2, 8, 12, 24, and 48 h were 1.89 and 2.84%, respectively, as illustrated in Table S6. All the results indicated that the chromatographic condition established could provide satisfying results and the standard solution was stable during a minimum of 48 h.

#### 3.6.3 Method Linearity Range and Sensitivity

As shown in Table 2, the calibration curves were plotted in the range of 10–500 mg/L and reconstituted daily for consecutive 4 days. The results showed accepted linearity with correlation coefficients (*R*^2^) over 0.9997, and the standard deviations of residuals were less than 3%. The slopes of the calibration curves were constant for 4 days demonstrating the excellent response stability of this method.

**Table 2:** Calibration Curves of the Linear Fitting

time	linear range (mg/L)	total data point	slope	intercept	coefficient *R*^2^
day 1	10–500	7	1.08 × 10^04^	–1.12 × 10^04^	0.9999
day 2	10–500	7	1.01 × 10^04^	–3.65 × 10^03^	0.9998
day 3	10–500	7	1.00 × 10^04^	–1.43 × 10^04^	0.9997
day 4	10–500	7	0.98 × 10^04^	–2.17 × 10^04^	0.9999

To further evaluate the LOD and LOQ of the developed method, ultrafiltration (MWCO = 10 kDa) was applied for the removal of proteins above 10 kDa (including the OPN) to obtain the blank matrix. By adding the OPN standard solution to the blank matrix resulting in a specific concentration, as shown in Figure S3, 3 times of signal-to-noise ratio (SNR) was defined as LOD and 10 times SNR for LOQ with 10 replicates (RSD < 20%). As a result, the measured LOD and LOQ were 0.04 and 0.13 mg/100 g, respectively.

#### 3.6.4 Accuracy (Recovery Rate)

To test the method's accuracy, the sample recovery rates were validated by testing the samples at three different concentrations (25, 75, and 250 mg/100 g). As illustrated in Figure S4 and Table S7, the sample recovery rates for these OPN levels were 107.8, 97.3, and 99.7% (*n* = 10) respectively, which could meet the relevant requirements of Council Directive 96/23/EC.

#### 3.6.5 Precision (Reproducibility)

Commercially available infant formula and raw milk were obtained, and their OPN contents were determined three times in 3 days. As the results summarized in Table 3 show, the OPN component could be found in all the samples of infant formula and raw milk, irrespective of whether OPN was fortified or not. The OPN contents in samples No. 1–6 were consistent with the natural concentrations in the raw material. The results from No. 7–12 showed that the products claiming OPN fortification had a higher concentration than that of theoretically calculated endogenous OPN. Nevertheless, sample No. 10 fortified with OPN only showed 1.7 times content compared to natural concentration, which indicated the proposed analytical strategy could be sensitive enough for product supervision. Results of the raw milk samples revealed that bovine and goat milks had similar OPN levels. In addition, all the 14 samples were analyzed three times (Day 1–3), and most of the RSDs were lower than 7.4%. After calculating with the Horwitz equation^30^ the method precision of OPN testing could satisfy the validation requirements for dairy products. These results indicated that this method was stable and reliable in determining the OPN content in real samples.

**Table 3:** Precision Test Results of OPN Content in the 14 Dairy Product Samples

no.	sample name	day 1, content (mg/100 g)	day 2, content (mg/100 g)	day 3, content (mg/100 g)	RSD (%)	compare to 100% human milk OPN level^33^^a^
1	22T067-stage 1	32.12	31.49	33.90	3.8	without extra OPN
2	22T087- stage 2	34.69	34.29	39.10	7.4	without extra OPN
3	22T088- stage 3	43.13	42.84	45.20	2.9	without extra OPN
4	22T035- stage 1	124.72	116.18	113.42	5.0	100%
5	22T083- stage 2	86.82	83.28	80.56	3.8	80%
6	22T034- stage 3	94.89	90.05	87.84	4.0	80%
7	22T078- stage 1	130.09	122.62	121.26	3.8	100%
8	22T081- stage 2	121.85	118.48	112.58	4.0	100%
9	22T082- stage 3	115.94	114.05	107.99	3.7	100%
10	22T037- stage 1	58.37	59.74	61.28	2.4	50%
11	22T079- stage 2	135.57	129.68	124.55	4.2	100%
12	22T080- stage 3	132.22	123.91	122.03	4.3	100%
13	22T010-cow’s milk	10.48	9.56	10.13	4.6	endogenous OPN
14	22T009-goat’s milk	9.93	10.21	9.54	3.4	endogenous OPN

a Compare to 100% human milk OPN level:

### 3.7 Method Comparison and Summary

Table 4 summarizes the chromatographic information, including analytes, standards, matrix types, LODs, and linear ranges. As presented, the ELISA method may be suitable for detecting liquid dairy products, although with a narrow linear range. The developed method in this work extended the applicability and linear range to 10–500 mg/L. Certain LC-MS methods were also sensitive, whereas the reproducibility and stability of detection were not ideal, especially for infant formula samples.^16,32,33,34^

**Table 4:** Comparison of Different OPN-Detecting Methods

method	principle	intact protein	standards for quantification	sample type	LOD	linear range	ref
ELISA	combination of antibody and antigen; colorimetric method	yes	recombinant human OPN	cell culture supernates, EDTA plasma, heparin plasma, urine, human milk	0.24 mg/L	0.3–20 mg/L	(35)
		yes	N-terminal milk OPN fragment	bovine milk	1.25 mg/L	0.4–67.8 mg/L	(36)
MS/MS	extraction, hydrolysis, and analysis of peptides	not intact	two peptides	milk products including powdered formula for infants and young children	10 mg/100 g	2–100 mg/L	(16)
		not intact	two peptides	bovine, buffalo, yak, goat, and sheep milk	2.0 mg/L	10–200 mg/L	(17)
UHPLC-DAD	extraction, ion-exchange chromatography	yes	bovine OPN, calibrated by AUC	powdered milk formula for infants and young children, cow’s milk, goat milk	0.04 mg/100 g	10–500 mg/L	this study

Compared to previous reports about the determination of the concentration of OPN on HPLC, this study exhibited obvious merits. The in-house OPN standard was determined by AUC, which solved the issue of lacking commercially available standards for accurate quantification. Moreover, the newly established method which quantifies intact OPN showed the advantages of direct determination, high sensitivity (0.04 mg/100 g), and simple operation. It can be expected that this method for the quantification and qualification of bovine OPN could provide guidance for studies on other milk proteins as well as inspire the determination of alternative proteins in the dairy industry.

## Supporting Information Available

Experiment: identification of OPN purity by mass balance calculation; UV absorption spectrogram of bovine OPN; SEC chromatogram of bovine OPN; Lowest detection limit result at 0.04 mg/mL; Recovery rates of OPN in milk samples (*n* = 10) at three different concentrations; Purity validation data by mass balance calculation wt %; Particle composition content analysis of OPN in the buffer of THAM; Particle composition content analysis of OPN with buffer of PBS; System suitability test #1 by the 25 mg/L standard (*n* = 6); System suitability test #2 by the 25 mg/L standard (*n* = 6); Stability test for the 25 mg/L standard (*n* = 6); Accuracy test (recovery rate) of OPN (*n* = 10) (PDF)
