# Supplementary material for: Analytical Ultracentrifugation-Calibrated Anion-Exchange Chromatography for Sensitive and Intact Determination of Osteopontin in Infant Formula and Dairy Products
Source: J Agric Food Chem. 2023 Sep 5;71(37):13880–8. doi: 10.1021/acs.jafc.3c03589 (PMC10515612; doi:10.1021/acs.jafc.3c03589)
Supplement: Supplementary file 1 — jf3c03589_si_001.pdf [file jf3c03589_si_001.pdf]

Supporting Information

Analytical ultra-centrifugation-calibrated anion-exchange chromatography for sensitive and intact determination of Osteopontin in infant formula and dairy products

Xiangxin Wang,[1] Dongying Cui,[1] Xueyin Qu,[2] Hong You, *[3] Fan Lei,[4] Jianqiao Li,[1]

Yang Xie, [1] Haoshu Zhang, [1] Yongjiu Zhang, [1] Shilong Jiang, [1] Qinggang Xie[*1]

1 Heilongjiang Feihe Dairy Co., Ltd., Beijing 100015, China

2 China Excellent Milk Academy (Tianjin) Co., Ltd. 300400, Tianjin

3 Eurofins US Food, 2200 Rittenhouse St Ste 175 Des Moines, IA, 50321-3155 United States.

4 Tsinghua University, State Key Laboratory of Membrane Biology, Beijing, 100084, China

* Co-Corresponding Author: Hong You, Email: hong.you@eurofinsus.com Tel: +1-5157081576.

* Co-Corresponding Author: Qinggang Xie, Email: xieqinggang@feihe.com Tel: +8610-6431766, Fax: +8610-6431766.

Experiment

Identification of OPN Purity by mass balance calculation

Residual water was determined by Karl Fischer Analysis based on the *Chinese Pharmacopoeia, 2020 Edition* (Four General Principles 0832, the First Law). The determination of total ash content by gravimetric method is based on the *Chinese Pharmacopoeia, 2020 Edition* (Part IV General Rule 2302). The mass balance calculation followed the equation below.

OPN purity %= $\frac{(100 - wt\%water - wt\%ash) \times ChromPurity}{100}$

Figures

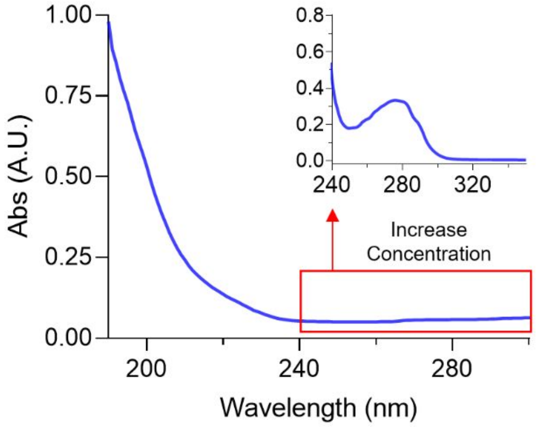

Figure S1 UV absorption spectrogram of bovine OPN.

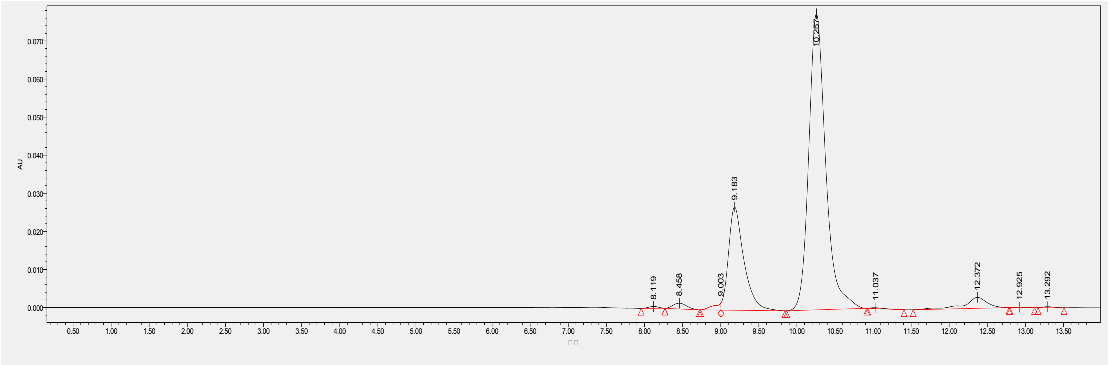

Figure S2 SEC chromatogram of bovine OPN.

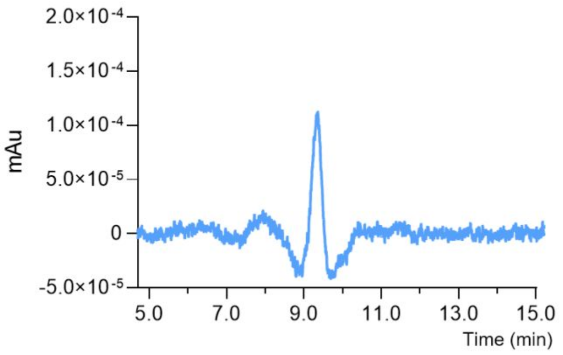

Figure S3 Lowest detection limit result at 0.04 mg/100g.

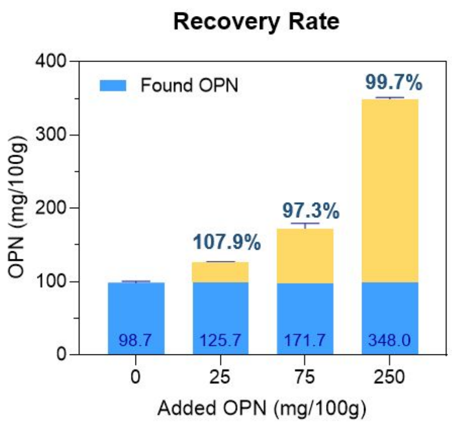

Figure S4 Recovery rates of OPN in milk samples (n=10) at three different concentrations.

Tables

Table S1. The purity validation data by mass balance calculation wt.%

Items	Water content %	Ash content %	Chromatogram Purity %	Actual Purity %
1	0.83	3.3	94.19	90.30
2	0.83	3.2	93.28	89.52
3	0.82	3.2	94.14	90.37
4	0.81	3.1	93.24	89.59
5	0.81	3.1	94.12	90.49
6	0.80	3.0	93.24	89.67
7	0.80	3.0	94.08	90.51
8	0.79	3.0	93.23	89.72
9	0.79	2.9	94.04	90.55
10	0.78	2.9	93.14	89.71
**AVE**	**0.81**	**3.07**	**93.67**	**90.04**
RSD	2.03	4.19	0.50	0.48

Table S2. Particle composition content analysis of OPN in the buffer of THAM

No.	Sedimentation co. (S)	M. W. (KD)	Aera (%)	Normalized content (wt.%)	Particle size (nm)
1	0.75	13.7	4.95%	5.29%	4.39
**2**	**1.508**	**39.3**	**84.91%**	**90.68%**	**6.22**
3	3.152	118.8	1.72%	1.84%	8.99
4	4.462	211.1	1.01%	1.08%	10.89
5	14.95	1227.2	1.05%	1.12%	19.58

Table S3. Particle composition content analysis of OPN with buffer of PBS

No.	Sedimentation co. (S)	M. W. (KD)	Aera (%)	Normalized content (wt.%)	Particle size (nm)
1	0.23	1.75	11.919	12.09	1.79
2	1.25	21.58	11.586	11.75	4.13
3	1.70	34.45	71.84	72.85	4.82
4	3.58	104.88	1.23	1.25	6.99
5	11.64	615.63	0.209	0.21	12.61
6	13.76	790.83	0.219	0.22	13.71
7	15.27	924.92	0.117	0.12	14.44
8	17.17	1102.12	0.155	0.16	15.31
9	19.47	1341.00	0.495	0.50	16.35
10	24.32	1858.57	0.845	0.86	18.23

Table S4. System suitability test #1 by the 25 mg/L standard (n = 6)

Injection Name<br>Selected Peak:	Ret. Time<br>(min)	Rel. Area<br>(%)	Area<br>(mAU*min)	Height<br>(mAU)
STD × Suitability#1 1	9.123	100	206423	7875
STD × Suitability#1 2	9.117	100	206301	7900
STD × Suitability#1 3	9.121	100	205463	7846
STD × Suitability#1 4	9.117	100	204939	7818
STD × Suitability#1 5	9.113	100	204340	7778
STD × Suitability#1 6	9.115	100	204279	7755
**RSD**	**0.04%**	**0.00%**	**0.46%**	**0.71%**

Table S5. System suitability test #2 by the 25 mg/L standard (n = 6)

Injection Name<br>Selected Peak:	Ret. Time<br>(min)	Rel. Area<br>(%)	Area<br>(mAU*min)	Height<br>(mAU)
STD × Suitability#2 1	9.119	100	201996	7611
STD × Suitability#2 2	9.121	100	200954	7574
STD × Suitability#2 3	9.117	100	200329	7510
STD × Suitability#2 4	9.118	100	208420	7991
STD × Suitability#2 5	9.121	100	207972	7987
STD × Suitability#2 6	9.124	100	207291	7933
**RSD**	**0.03%**	**0.00%**	**1.85%**	**2.90%**

Table S6. Stability test for the 25 mg/L standard (n = 6)

Intervals (h)	Injection Name Selected Peak:	Ret. Time (min)	Rel. Area (%)	Area (mAU*min)	Height (mAU)
0	STD × Stability 1	9.111	100	203455	7724
2	STD × Stability 2	9.116	100	202233	7669
8	STD × Stability 3	9.124	100	211663	8199
12	STD × Stability 4	9.125	100	210214	8128
24	STD × Stability 5	9.122	100	209827	8113
48	STD × Stability 6	9.123	100	209433	8061
	**RSD**	**0.06%**	**0.00%**	**1.89%**	**2.84%**

Table S7. Accuracy test (recovery rate) of OPN (n=10)

No.	Samples	Standard addition level (mg/100g)	Content mg/100g	Average mg/100g	Recovery Rate %	RSD%
1	22T034-1	0	101.78	98.72	-	1.95
2	22T034-2		98.25			
3	22T034-3		96.53			
4	22T034-4		99.57			
5	22T034-5		99.22			
6	22T034-6		96.66			
7	22T034-7		99.93			
8	22T034-8		95.88			
9	22T034-9		98.61			
10	22T034-10		100.77			
11	22T034-1	25	122.98	125.69	107.8	1.27
12	22T034-2		125.38			
13	22T034-3		127.04			
14	22T034-4		124.57			
15	22T034-5		126.52			
16	22T034-6		125.30			
17	22T034-7		127.85			
18	22T034-8		127.73			
19	22T034-9		124.03			
20	22T034-10		125.50			
21	22T034-1	75	173.26	171.73	97.3	4.42
22	22T034-2		167.04			
23	22T034-3		174.72			
24	22T034-4		169.51			
25	22T034-5		163.62			
26	22T034-6		169.77			
27	22T034-7		166.81			
28	22T034-8		190.90			
29	22T034-9		167.96			
30	22T034-10		173.67			
31	22T034-1	250	341.68	348.01	99.7	0.96
32	22T034-2		349.40			
33	22T034-3		347.94			
34	22T034-4		348.42			
35	22T034-5		352.71			
36	22T034-6		347.37			
37	22T034-7		343.51			
38	22T034-8		351.85			
39	22T034-9		348.06			
40	22T034-10		349.17			
